# Application of extracorporeal shock wave therapy in pain management: a bibliometric analysis of current advances and trends

**DOI:** 10.3389/fmed.2026.1867854

**Published:** 2026-07-17

**Authors:** Xin Zhao, Pan Hou, Zhengtong Qiao, Kai Yang, Feng Gu, Yapeng Li, Yanan Feng, Zhijie Zhang

**Affiliations:** Rehabilitation Therapy Center, Luoyang Orthopedic-Traumatological Hospital, Henan Provincial Orthopedic Hospital, Luoyang, China

**Keywords:** bibliometric analysis, cross-database validation, extracorporeal shock wave therapy, musculoskeletal pain, pain management

## Abstract

**Objectives:**

This study aimed to map the global research landscape, emerging topics, key contributors, and developmental trends in extracorporeal shock wave therapy (ESWT) for pain management.

**Methods:**

English-language publications on ESWT for pain management published from January 1, 2000, to December 31, 2025, were retrieved from the Web of Science Core Collection (WoSCC), with PubMed used for independent validation. Using software such as Excel, VOSviewer, RStudio, CiteSpace, BICOMB, and Incites, we conduct visual analyses of basic literature information, publication patterns, output trends, countries/regions, institutions, journals, keywords, and influential authors.

**Results:**

Of 1,520 records identified, 774 WoSCC articles met the inclusion criteria. Research output increased substantially over time and involved 57 countries or regions. China and Italy were the most productive countries, whereas Germany showed the highest citation impact. Maffulli N was the most prolific author, and Rompe JD ranked first in citation frequency and h-index. Clinical Orthopedics and Related Research was the most frequently cited journal. Keyword and citation analyses identified major themes including plantar fasciitis, shoulder disorders, low back pain, lateral epicondylitis, chronic pelvic pain syndrome, knee osteoarthritis, core decompression, and myofascial pain syndrome. Musculoskeletal pain remained the dominant focus, whereas knee osteoarthritis, chronic pelvic pain syndrome, and myofascial pain syndrome emerged as growing topics. PubMed validation including 555 articles showed comparable publication trends and core research themes.

**Conclusion:**

ESWT research in pain management has progressed from early efficacy validation toward translational expansion, mechanistic investigation, regenerative applications, and broader clinical indications. Cross-database validation supports the robustness of the WoSCC-based findings. Future studies should prioritize multicenter randomized trials, standardized treatment protocols, mechanistic exploration, and international collaboration to strengthen the physiological and clinical evidence base for ESWT as a non-pharmacological pain-management strategy.

## 1 Introduction

Pain, especially chronic musculoskeletal pain, represents a major global public health challenge. Its high prevalence and associated disability substantially impair patients' quality of life and impose a considerable burden on healthcare systems (1). In 2019, the World Health Organization officially classified chronic pain as an independent disease category in the International Classification of Diseases, 11th Revision (ICD-11). In 2020, the International Association for the Study of Pain (IASP) updated its pain classification criteria (2, 3). Together, these developments underscore the urgent need for standardized and diversified approaches to pain diagnosis and treatment. In the context of the global opioid crisis, non-pharmacological analgesic therapies have become a major focus of clinical research and practice because of their favorable safety profiles. Extracorporeal shock wave therapy (ESWT) is among the most promising physical therapy modalities in this field (4–7).

Owing to its non-invasiveness, high precision, and low complication rate, ESWT has been increasingly applied in the treatment of plantar fasciitis (8, 9), lateral epicondylitis (10, 11), knee osteoarthritis (12, 13), chronic pelvic pain syndrome (14, 15), and other pain-related conditions. Its mechanisms of action involve the regulation of local tissue metabolism, attenuation of inflammatory responses, and promotion of tissue repair and regeneration through mechanical stress and cavitation effects. These mechanisms have been preliminarily supported by multiple clinical studies. In recent years, advances in rehabilitation medicine and sports medicine have led to a growing body of research on ESWT for pain management. In physiotherapy and rehabilitation practice, ESWT is also used by physical therapists as a non-invasive adjunctive modality for pain relief and functional recovery in selected musculoskeletal conditions, particularly plantar heel pain and tendinopathy (16, 17). The research focus has gradually shifted from evaluating the efficacy of ESWT for individual conditions to broader areas, including optimization of combination therapies, mechanistic investigation, and expansion of clinical indications. International collaboration and multidisciplinary integration have also become increasingly evident (4, 18, 19).

Although ESWT has been widely recognized for pain management, existing studies have mainly focused on clinical efficacy in specific conditions, leaving the overall research landscape, hotspot evolution, collaboration patterns, and future directions insufficiently understood. Therefore, a bibliometric analysis is necessary to quantitatively and visually map the development of this field. In this study, we used the Web of Science Core Collection (WoSCC) as the primary data source, supplemented by PubMed for cross-database validation, to systematically analyze global ESWT-related pain research from 2000 to 2025. This study aimed to identify research trends, hotspot topics, academic collaboration patterns, and potential future directions, thereby providing evidence to support clinical optimization and research innovation.

## 2 Materials and methods

### 2.1 Data sources and literature search

Literature records were retrieved from WoSCC and PubMed for the period from January 1, 2000, to December 31, 2025. All searches and data downloads were completed on the same day to minimize discrepancies caused by daily database updates. The primary dataset for bibliometric mapping and visualization was obtained from the SCI-E in WoSCC. The WoSCC search strategy was as follows: TC = (“Extracorporeal Shock Wave Therapy” OR “Therapy, Shock Wave” OR “Shock Wave Therapy” OR “Shock Wave Therapies” OR “Shockwave Therapies, Extracorporeal” OR “Therapy, Extracorporeal Shockwave” OR “Extracorporeal Shockwave Therapies” OR “Shockwave Therapy, Extracorporeal”) AND TC = (“chronic pain” OR “shoulder pain” OR “neck pain” OR “low back pain” OR “musculoskeletal pain” OR “musculoskeletal diseases” OR “fibromyalgia” OR “arthritis” OR “osteoarthritis” OR “rheumatoid arthritis” OR “spondylarthritis” OR “ankylosing spondylitis” OR “widespread pain” OR “knee pain” OR “hip pain” OR “ankle pain” OR “epicondylalgia” OR “musculoskeletal disorders” OR “osteoarthrosis” OR “analgesia” OR “pain” OR “acute pain” OR “pelvic pain”). To assess the robustness of the findings and reduce potential database-specific bias, a parallel validation dataset was retrieved from PubMed using a conceptually equivalent search strategy that combined Medical Subject Headings (MeSH) with title/abstract keywords: ((“Extracorporeal Shockwave Therapy”[MeSH] OR “Extracorporeal Shock Wave Therapy”[tiab] OR “Shock Wave Therapy”[tiab] OR “Shockwave Therapy”[tiab]) AND (“Pain”[MeSH] OR “Chronic Pain”[MeSH] OR “Musculoskeletal Pain”[MeSH] OR “Low Back Pain”[MeSH] OR “Shoulder Pain”[MeSH] OR “Knee Pain”[MeSH] OR “Pelvic Pain”[MeSH] OR “Osteoarthritis”[MeSH] OR “Fibromyalgia”[MeSH] OR pain[tiab] OR “chronic pain”[tiab] OR “shoulder pain”[tiab] OR “neck pain”[tiab] OR “low back pain”[tiab] OR “musculoskeletal pain”[tiab] OR “musculoskeletal diseases”[tiab] OR fibromyalgia[tiab] OR arthritis[tiab] OR osteoarthritis[tiab] OR “rheumatoid arthritis”[tiab] OR spondylarthritis[tiab] OR “ankylosing spondylitis”[tiab] OR “widespread pain”[tiab] OR “knee pain”[tiab] OR “hip pain”[tiab] OR “ankle pain”[tiab] OR epicondylalgia[tiab] OR “musculoskeletal disorders”[tiab] OR osteoarthrosis[tiab] OR analgesia[tiab] OR “acute pain”[tiab])).

### 2.2 Data processing

#### 2.2.1 Inclusion criteria

The inclusion criteria were as follows: (1) document type: article or review; (2) language: English; (3) studies related to extracorporeal shock wave therapy for pain management; (4) complete bibliographic information, including title, country, authors, keywords, and source journal; and (5) publication year between 2000 and 2025.

#### 2.2.2 Exclusion criteria

The exclusion criteria were as follows: (1) studies unrelated to the topic that only mentioned extracorporeal shock wave therapy or pain; (2) animal-only studies without human data; and (3) duplicate publications.

#### 2.2.3 Data standardization

The retrieved records were exported in plain-text format with full records and cited references. Keywords, countries or regions, and institutional names were standardized using the data import and export functions of CiteSpace. Two researchers independently screened the titles, abstracts, and keywords to exclude irrelevant studies. The relevance of the remaining records was further confirmed by full-text review. Disagreements were resolved through consultation with a third reviewer. To ensure data consistency, institutional names, countries or regions, and keywords were systematically standardized. Institutional names were standardized according to their official full names, and synonymous keywords were merged. To improve the reliability of the author-level analysis, author names were first converted into a consistent format of “Last Name, First Name.” When an author appeared under different name forms, such as “Rompe, J. D.” and “Rompe JD,” a customized string-processing procedure in R was applied to identify the most frequently used form and adopt it as the standardized entry. The full set of merged author-name variants is listed in Supplementary Table S1. Institutional affiliations were retrieved from the address information, after which non-essential elements, including postal codes and department-level details, were excluded. The remaining affiliation names were harmonized according to a manually compiled thesaurus. For instance, “Sapienza University Rome” and “University Roma La Sapienza” were unified as “University Roma La Sapienza.” The complete institutional synonym list is presented in Supplementary Table S2. Keywords were also normalized using a pre-defined synonym dictionary, which enabled the integration of equivalent terms and spelling variations. For example, “shock wave therapy” was consolidated under “extracorporeal shock wave therapy,” while “randomized controlled-trial” was unified as “randomized controlled trial.” Further details are provided in Supplementary Table S3. For country- and region-level analyses, geographic names were likewise harmonized. Publications from “Taiwan” were grouped under China to maintain consistency with the geographic scope of the study, and this classification was not intended to convey any political position. The detailed list of consolidated countries and regions is shown in Supplementary Table S4.

### 2.3 Research methods and data analysis

Excel (version 16.0) was used to analyze annual publication volumes; VOSviewer (version 1.6.20) was used to visualize collaborations among countries, institutions, and authors; RStudio (R version 4.2.2) and the bibliometrix package were used to visualize journal publications, countries, cited authors, and keyword clouds; CiteSpace (version 6.4.R1) was used to visualize cited references, generate keyword cluster maps, perform emerging term analysis, and track temporal evolution. To ensure data quality prior to formal analysis, all bibliometric records retrieved were pre-processed, including manual standardization of author names and institutional affiliations to resolve inconsistencies. Thresholds for the number of publications and citations were determined based on the characteristics of the data distribution to ensure that meaningful representative structures were highlighted while guaranteeing sufficient inclusion of nodes, thereby ensuring the robustness of the network analysis results.

The VOSviewer parameter settings are as follows: the analysis type was set to “co-authorship”, the analysis units were set to “Authors/Organizations/Countries” and the “full count” method was used. Standardization was performed using the association strength method. In the visualization configuration, node weights represent the number of publications, and cluster analysis is used to identify different collaboration clusters. The parameter settings for CitesSpace 6.4.R1 are as follows: (1) time slices (2000 to 2025), with each slice covering 1 year; (2) when selecting node types, “keyword” and “reference” are chosen; (3) for association strength, “cosine” is selected, and for scope, “within slices” is selected. (4) We used the g-index as the screening criterion, with a *k*-value of 25. Journal impact factors were retrieved from the Journal Citation Reports^®^ (JCR) 2025 edition to contextualize citation influence.

We used CiteSpace software to perform keyword visualization analysis on the converted dataset files and simultaneously calculated the centrality of keyword nodes. The higher the node centrality value, the more significant the role that node plays in the visualized network. Additionally, in the author visualization analysis, we conducted analysis based on Price's Law. The publication count of core authors must satisfy the condition:*M*_*p*_ (where $M_{p}=0.749\times^{*}\sqrt{N_{pmax}}$), *M*_*p*_ represents the minimum publication count for an author, and *N*_*pmax*_ represents the number of papers by the most prolific author. Finally, in the keyword clustering analysis, the LLR algorithm was applied to generate a keyword clustering view. The LLR algorithm is provided by CiteSpace for clustering and tag extraction, and the software performs the clustering analysis automatically. Modality (*Q* value) and average contour width (*S* value) were used to evaluate clustering performance. Generally, Q > 0.3 indicates a significant clustering structure, whereas S > 0.5 indicates acceptable cluster homogeneity.

## 3 Results

### 3.1 Literature search and screening results

**Figure 1:**
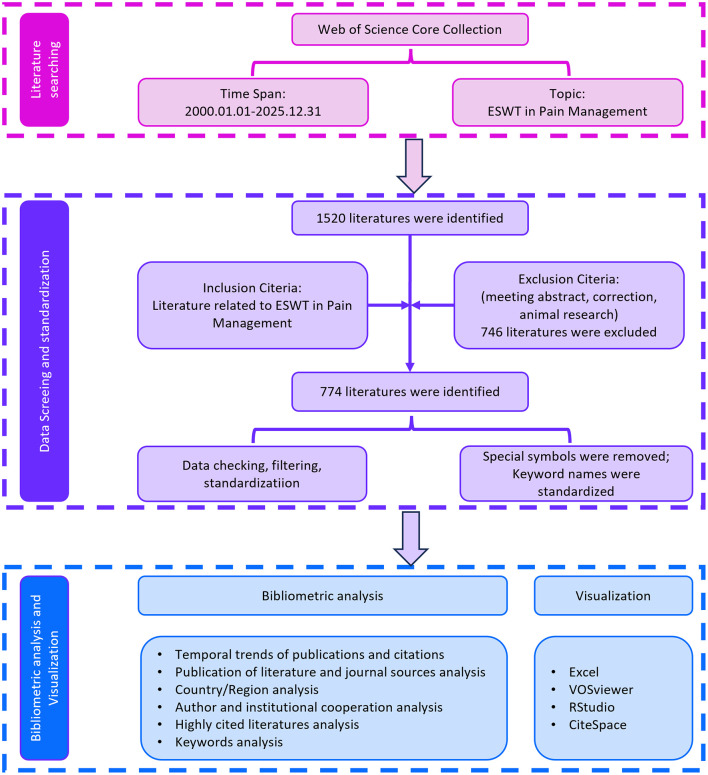
Literature data screening process and technical roadmap.

The initial search identified 1,520 records. After duplicates, irrelevant studies, and animal-only studies were excluded, 774 eligible studies were included in the final analysis. The detailed screening process is shown in Figure 1.

### 3.2 Analysis of annual publication trends and journal sources

**Figure 2:**
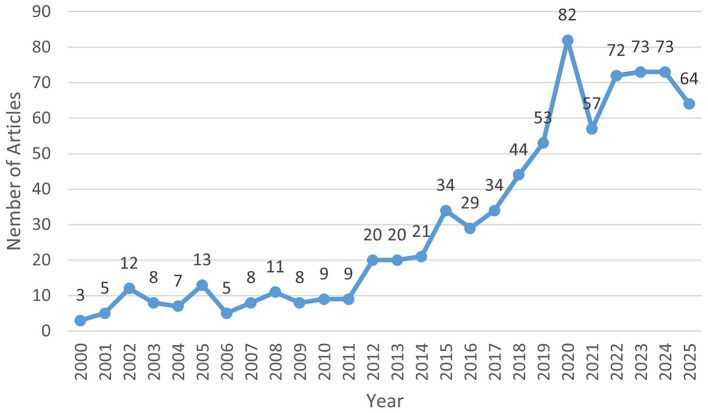
Annual distribution of publications.

As shown in Figure 2, the number of publications on extracorporeal shock wave therapy for pain management showed an overall upward trend. From 2000 to 2011, the annual publication output remained low and showed only minor fluctuations. Beginning in 2012, a gradual upward trend emerged. This upward trend aligns with increased clinical attention to pain management and successive international initiatives regarding the classification of chronic pain and education on pain medications. Since 2015, the growth in publications has accelerated significantly, with the annual number of published papers exceeding 60 and peaking at 82 between 2020 and 2023. This turning point coincided with an aging population, rising clinical demand for non-pharmacological pain relief alternatives against the backdrop of the global opioid crisis, and the 2020 update to the pain classification criteria by the International Association for the Study of Pain (IASP). These policy and guideline updates occurred simultaneously with the expansion of academic output in this field; however, a causal relationship cannot be fully confirmed based solely on bibliometric observations.

**Figure 3:**
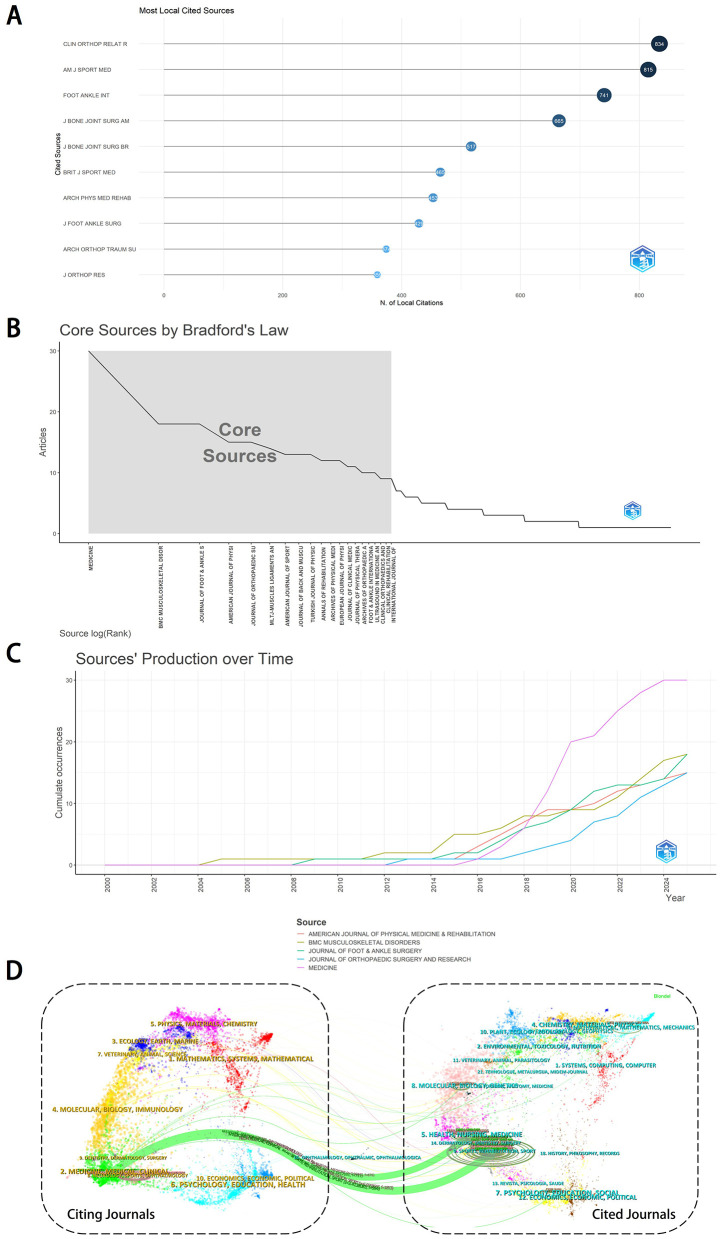
Visual analysis of publication volume and journal sources. **(A)** Top 10 journals by citation count; **(B)** Core journals identified based on Bradford's Law; **(C)** Annual output trends for the top 5 journals by publication volume; **(D)** Journal citation relationship network (including clustering of citing and cited journals).

A total of 318 journals published relevant studies, among which *MEDICINE* had the highest publication output, with 30 articles (Table 1). *CLINICAL ORTHOPEDICS AND RELATED RESEARCH* had the highest citation count, with 834 citations (Figure 3A). As a leading journal in orthopedics, *CLINICAL ORTHOPEDICS AND RELATED RESEARCH* focuses on clinical treatment and rehabilitation of orthopedic diseases. Together with Medicine, it represents an important publication and citation source in this field. According to Bradford's law, 20 core journals, led by *MEDICINE, BMC MUSCULOSKELETAL DISORDERS*, and *JOURNAL OF FOOT & ANKLE SURGERY*, accounted for approximately 28% of the total publications (Figure 3B).

**Table 1:** The top 10 journals ranked by the counts of publications.

Rank	Journal	Publication	Cited frequency	Country	IF (2025)	h_index
1	Medicine	30	271	United States	2.0	12
2	BMC musculoskeletal disorders	18	343	England	2.8	10
3	Journal of foot and ankle surgery	18	429	United States	1.4	10
4	American journal of physical medicine and rehabilitation	15	272	United States	2.3	9
5	Journal Of orthopedic surgery and research	15	199	England	3.8	7
6	MLTJ-muscles ligaments and tendons journal	14	90	Italy	1.1	5
7	American journal of sports medicine	13	815	United States	5.4	13
8	Journal Of back and musculoskeletal rehabilitation	13	142	Netherlands	1.6	6
9	Turkish journal of physical medicine and rehabilitation	13	48	Turkey	1.3	5
10	Annals Of rehabilitation medicine-arm	12	272	South Korea	2.8	9

As shown in Figure 3C, before 2015, traditional orthopedic journals such as *ARCHIVES OF ORTHOPEDIC AND TRAUMA SURGERY* and *FOOT & ANKLE INTERNATIONAL* had the highest publication output. After 2015, the output of *MEDICINE, BMC MUSCULOSKELETAL DISORDERS*, and *JOURNAL OF FOOT & ANKLE SURGERY* increased rapidly and remained among the leading journals, suggesting a shift toward multidisciplinary integration and clinical application. The dual-map journal overlay (Figure 3D) illustrates citation relationships between citing and cited journals, with citing journal clusters shown on the left and cited journal clusters shown on the right. The green citation paths indicate that studies published in journals from the fields of health, nursing, and medicine; dermatology, dentistry, and surgery; and psychology, education, and social sciences were most frequently cited by journals in dentistry, dermatology, and surgery; medicine, medical, and clinical research; and neurology, sports, and ophthalmology. The yellow citation paths indicate that studies published in molecular biology and genetics journals were most frequently cited by journals in molecular biology and immunology, as well as veterinary and animal science.

### 3.3 Visualization analysis of international collaboration

**Figure 4:**
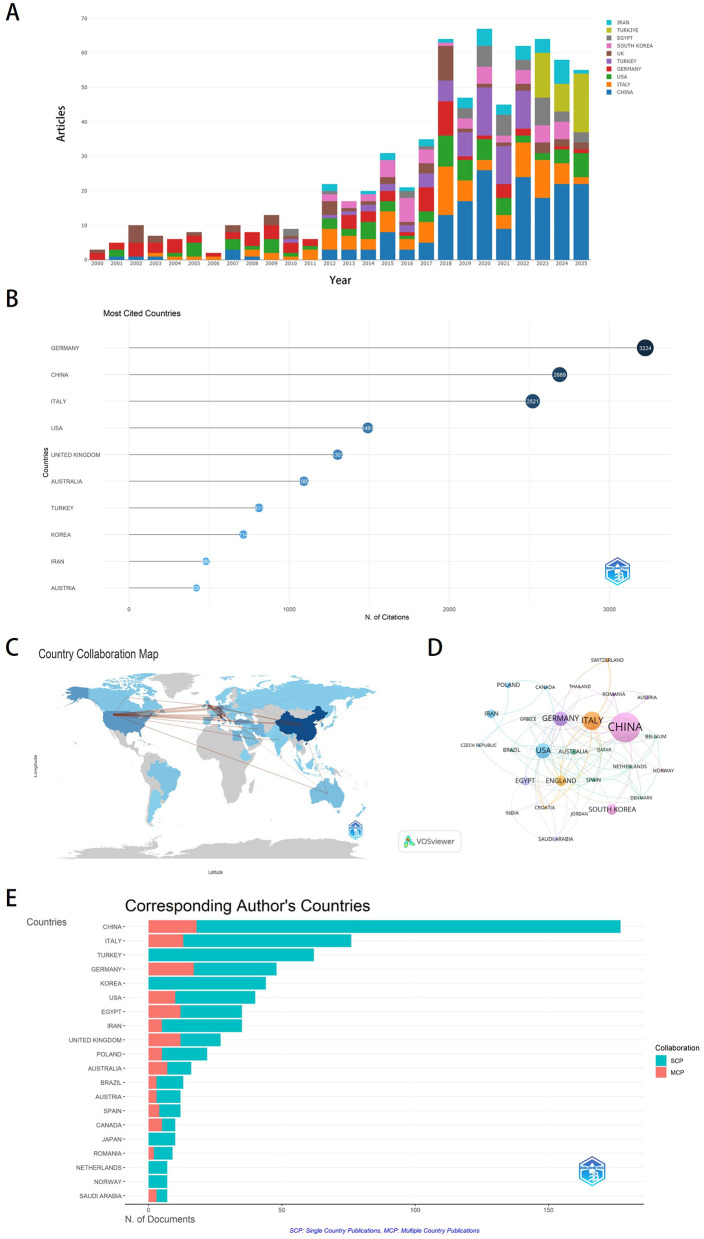
Visualization of international collaboration in scientific publications. **(A)** Distribution of publications by country; **(B)** Top 10 countries by citation frequency; **(C)** Distribution of international collaboration; **(D)** Network of international collaboration; **(E)** Top 10 countries of corresponding authors.

ESWT-related pain studies involved authors from 57 countries. The top 10 countries by publication output were China (507), Italy (279), Turkey (162), the United States (161), Germany (154), South Korea (117), Egypt (103), Iran (92), the United Kingdom (89), and Australia (67; Figure 4A). In terms of citation frequency, Germany ranked first with 3,224 citations, followed by China (2,689 citations) and Italy (2,521 citations; Figure 4B). In the inter-country collaboration map, thicker lines indicate stronger collaborative links. China showed close collaboration with Germany, Italy, the United States, and Australia, whereas the United States collaborated frequently with Germany, the United Kingdom, Canada, and other countries (Figures 4C, D).

Single-country publications (SCPs) were defined as articles in which all authors were affiliated with institutions in the same country, whereas multi-country publications (MCPs) were defined as articles involving authors from more than one country and were used to represent international collaboration. Based on the MCP proportions, Canada showed the highest level of international collaboration (50.0%), followed by the United Kingdom (44.4%) and Germany (35.4%). Among the 177 articles with corresponding authors from China, only 18 (10.2%) were MCPs, indicating that domestic collaboration pre-dominated and that international collaboration could be further strengthened (Figure 4E).

In addition to publication output, citation impact, and international collaboration patterns, we further examined the socioeconomic distribution of contributing economies. All economies were categorized based on the World Bank fiscal year 2026 income classification, which relies on 2024 Atlas GNI per capita data. In total, 57 economies contributed to ESWT-related pain research. Among them, 56 could be assigned to standard income tiers: 36 (64.29%) high-income economies, 13 (23.21%) upper-middle-income economies, six (10.71%) lower-middle-income economies, and one (1.79%) low-income economy. The remaining one economy, Ethiopia, was marked as temporarily unclassified in the FY26 World Bank classification due to the absence of credible 2024 GNI per capita estimates. These findings suggest that research on ESWT for pain management was more frequently represented in high-income and upper-middle-income economies than in lower-middle-income and low-income economies.

### 3.4 Visual analysis of author and institutional collaboration

**Figure 5:**
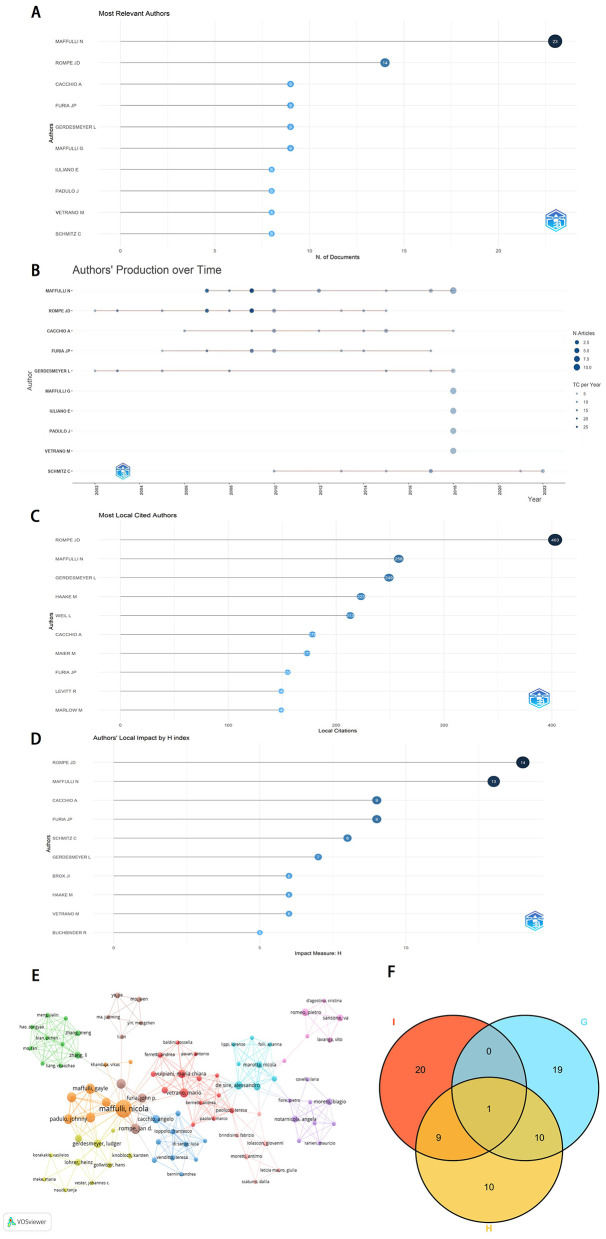
Visualization of authors. **(A)** Top 10 authors by publication count; **(B)** Annual publication trends of the top 10 authors (each point represents an author's publications at a specific point in time; point size indicates publication count, and point color indicates citation frequency); **(C)** Top 10 authors by citation frequency; **(D)** Top 10 authors ranked by h-index; **(E)** Author collaboration network diagram (larger font size indicates higher publication volume; thicker lines indicate closer collaboration); **(F)** Author Venn diagram.

The 774 included publications involved 3,188 authors. Among the top 10 authors ranked by publication output, MAFFULLI N ranked first with 23 publications, followed by ROMPE JD (14), CACCHIO A (9), and FURIA JP (9). The publication output and annual publication trends of the top 10 authors are shown in Figures 5A, B. Among the top 10 authors ranked by total citations, ROMPE JD ranked first with 403 citations, followed by MAFFULLI N (258 citations), GERDESMEYER L (249 citations), and HAAKE M (223 citations) (Figure 5C). Among the top 10 authors ranked by h-index, ROMPE JD ranked first with an h-index of 14, followed by MAFFULLI N (h-index = 13), CACCHIO A (h-index = 9), and FURIA JP (h-index = 9; Figure 5D).

Based on Price's law, Mp was calculated as 3.59. Therefore, authors with four or more publications were defined as core authors, resulting in the identification of 81 core authors. The author collaboration network is shown in Figure 5E. Each node represents an author, and node size indicates publication output. More connections between nodes indicate closer collaboration, whereas different colors represent distinct collaboration clusters. MAFFULLI, NICOLA from The London Independent Hospital, London, United Kingdom, showed the highest connectivity and collaborated closely with researchers from Wholelife Clinic, London, United Kingdom; University eCampus, Novedrate, Italy; and the OrthoTrauma Evaluation Institute, Mainz, Germany. These researchers collaborated on studies evaluating the short- and long-term efficacy of ESWT in patients with patellar tendinopathy.

Analysis of core authors, highly collaborative authors, and highly cited authors helps identify the major contributors and research characteristics of this field. Accordingly, core authors were labeled as “H,” the top 30 most collaborative authors as “G,” and the top 30 most cited authors as “I.” Venn diagram analysis showed that one author belonged to all three categories (H, G, and I), nine authors belonged to both the I and H categories, and 10 authors belonged to both the H and G categories. In addition, 10 authors belonged only to the H category, 20 only to the I category, and 19 only to the G category (Figure 5F).

**Figure 6:**
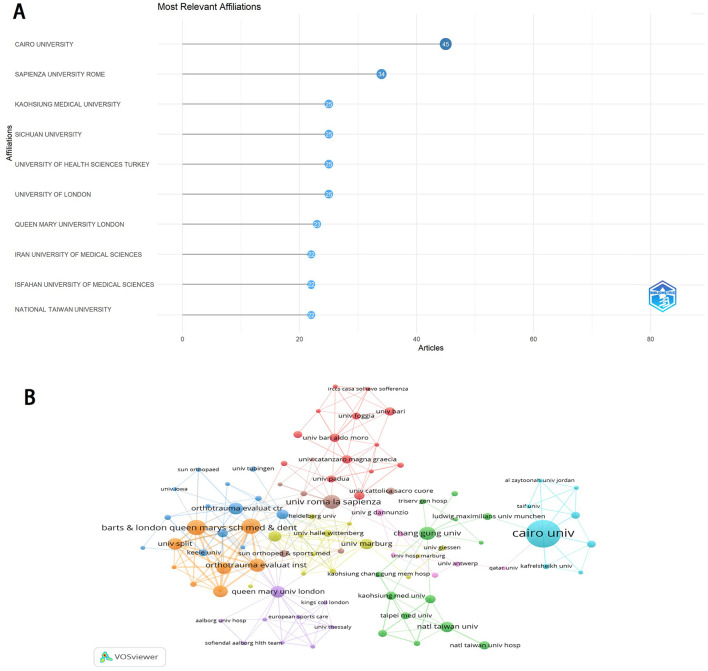
Visualization of institutions. **(A)** Top 10 institutions by publication volume; **(B)** Institutional collaboration distribution diagram.

A total of 818 institutions contributed to publications on extracorporeal shock wave therapy for pain management. The top 10 institutions accounted for 34.63% of all publications, with the CAIRO UNIVERSITY contributing the highest number of publications (*n* = 45; Figure 6A). Betweenness centrality (BC) was used to measure the importance of nodes within the collaboration network. In the institutional collaboration network, each node represents an institution, larger labels indicate higher publication output, and connecting lines represent inter-institutional collaboration. The University of Munich had the highest betweenness centrality. As shown in Figure 6B, close collaboration was observed among institutions including the University of London, Queen Mary University of London, Barts Health NHS Trust, Mile End Hospital, and the University of Salerno.

### 3.5 Citation analysis

**Figure 7:**
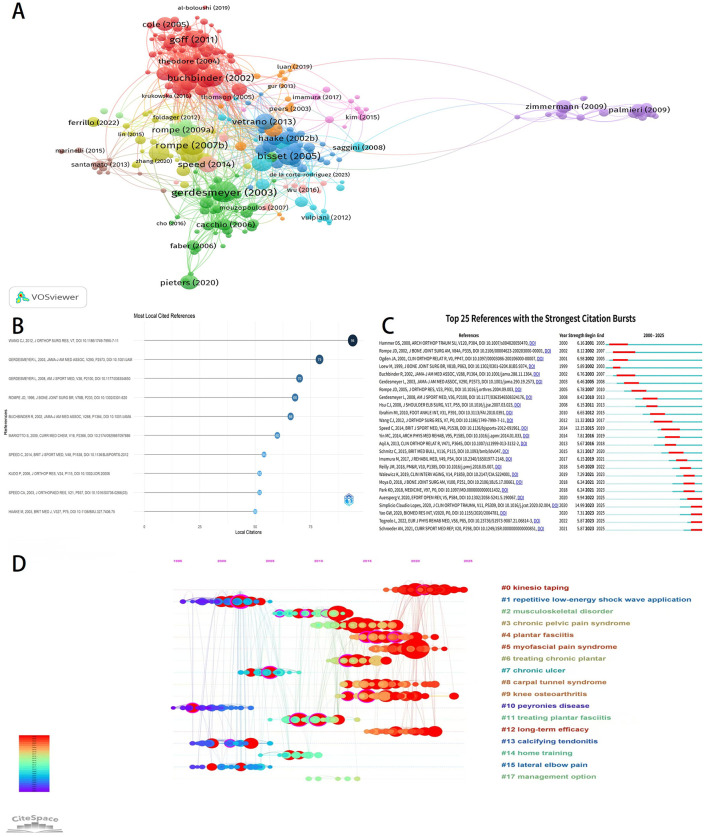
Visualization of cited literature. **(A)** Analysis of thematic coupling in research literature (node size indicates the number of citations; edges represent collaborative relationships (cited by the same literature); colors represent clusters of cited authors); **(B)** Top 10 most-cited papers; **(C)** Emergence analysis of cited literature; **(D)** Timeline analysis of cited literature.

As shown in Figure 7A, bibliographic coupling analysis identified eight thematic clusters, all of which were related to the application of extracorporeal shock wave therapy in the treatment of disease-related pain. The purple cluster mainly involved chronic disorders of the male urogenital system, particularly Peyronie's disease and chronic pelvic pain syndrome. The red cluster was related to plantar fasciitis; the green cluster to shoulder tendon disorders; the yellow cluster to lower-limb tendon disorders; the blue cluster to patellar tendinopathy; the orange cluster to myofascial pain syndrome; the pink cluster to knee osteoarthritis; and the brown cluster to spasticity and neuropathic pain. The top 10 most-cited references are shown in Figure 7B. The three most-cited references were as follows: a 2012 review by WANG CJ published in Journal of Orthopedic Surgery and Research with 94 citations, which reported that ESWT is a non-invasive and effective treatment for various musculoskeletal disorders, including plantar fasciitis and lateral epicondylitis; a 2003 randomized controlled trial by Gerdesmeyer et al. published in JAMA with 79 citations, which demonstrated the efficacy of ESWT for chronic calcific rotator cuff tendinitis and showed that high-energy therapy was superior to low-energy therapy; and a 2008 randomized controlled trial by Gerdesmeyer et al. published in The American Journal of Sports Medicine with 70 citations, which showed that radial ESWT safely and effectively improved pain, function, and quality of life in patients with chronic refractory plantar fasciitis. Among the top 10 most-cited articles, five focused on the treatment of plantar fasciitis.

Citation burst analysis clearly illustrated the temporal evolution of research trends in ESWT (Figure 7C). The top 25 references with the strongest citation bursts mainly focused on validating the clinical efficacy of ESWT and expanding its applications in musculoskeletal disorders. HAMMER 2000, ROMPE 2002, and GERDESMEYER 2003 reported the therapeutic effects of ESWT on plantar fasciitis, calcific shoulder tendinitis, and lateral humeral epicondylitis, respectively. These studies represented early clinical validation of ESWT for specific indications and attracted considerable attention. WANG CJ 2012 and SPEED 2014 shifted the research focus toward systematic evidence synthesis and mechanistic exploration, while extending ESWT applications to other indications, such as avascular necrosis of the femoral head. AUERSPERG 2020 and SIMPLICIO CLAUDIO LOPES 2020 further addressed the long-term efficacy of ESWT for musculoskeletal pain conditions, optimization of treatment protocols, and application to new indications. These studies suggest that the field has evolved from initial efficacy confirmation to refinement of treatment strategies and deeper mechanistic investigation. As shown in Figure 7D, citation clustering analysis showed a mean silhouette value (S) of 0.9199 and a modularity value (Q) of 0.8073, indicating a significant and reliable clustering structure. Clusters 2, 4, 5, 6, 8, 9, 13, and 15 primarily involved musculoskeletal conditions, including plantar fasciitis, carpal tunnel syndrome, knee osteoarthritis, myofascial pain syndrome, tendinopathy, and lateral epicondylitis. Clusters 3, 7, and 10 were related to chronic pelvic pain, chronic ulcers, and Peyronie's disease. Clusters 0, 14, and 17 represented adjunctive or comprehensive management strategies associated with ESWT, including kinesiology taping, home exercise, and other holistic treatment approaches.

### 3.6 Visual analysis of keywords

**Figure 8:**
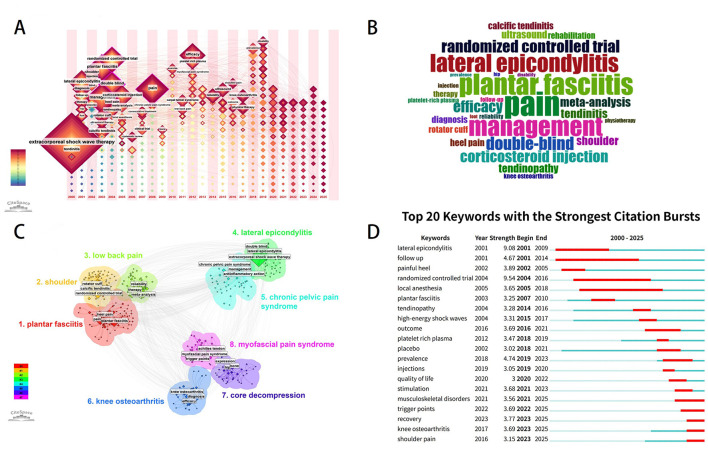
Visual analysis of keywords. **(A)** Keyword timeline; **(B)** Word cloud of keywords from local clusters; **(C)** Co-occurrence map of keyword clusters; **(D)** Top 25 keywords by emergence intensity.

The keyword timeline (Figure 8A) illustrates the evolution of research trends in the field of ESWT over time. Research in this field over the past two decades can be divided into three phases: Phase I (2000–2007), an exploratory phase focusing on the preliminary validation of efficacy for conditions such as “tennis elbow” and “heel pain”; Phase II (2008–2016), a period of methodological standardization, during which “randomized controlled trials” became the dominant research feature, and “plantar fasciitis” and “tendinopathy” emerged as core disease models; and Phase III (2017–present), the expansion and integration phase, with research shifting toward “platelet-rich plasma” (combination therapy), “prevalence,” “quality of life,” and “musculoskeletal disorders,” reflecting a broader focus from analgesic efficacy to epidemiology, functional rehabilitation, and comprehensive health outcomes. High-frequency keyword analysis (Figure 8B) supports these focal points, with terms such as “plantar fasciitis,” “shoulder disorders,” and “randomized controlled trials” appearing frequently. The keyword clustering network (Figure 8C) formed seven distinct clusters [clustering silhouette (S) = 0.7609, modularity (Q) = 0.3877], indicating significant and reliable clustering. These clusters included #0 plantar fasciitis, #1 shoulder disorders, #3 tennis elbow, and others. Among these, #0 and #1 serve as core nodes, widely connected to other clusters, forming the central research framework of this field. Emergence analysis (Figure 8D) reveals the strong rise of themes such as “randomized controlled trials” (2004–2016) and more recently, “quality of life” and “recovery,” reflecting the field's evolution from validating therapeutic efficacy to optimizing long-term management.

### 3.7 Cross-database validation with PubMed

To evaluate the robustness of our main findings and reduce potential bias arising from reliance on a single database, we performed cross-database validation using an independent dataset retrieved from PubMed. After screening, PubMed yielded 555 publications, whereas the WoSCC dataset included 774 publications. This numerical difference likely reflects the distinct scopes of the two databases: WoSCC provides broader coverage of interdisciplinary research, basic science, and high-impact international journals, whereas PubMed focuses primarily on clinical and biomedical literature, including regional clinical journals and early-stage studies. Despite these differences, the comparative analysis of key bibliometric indicators showed a high degree of consistency.

**Figure 9:**
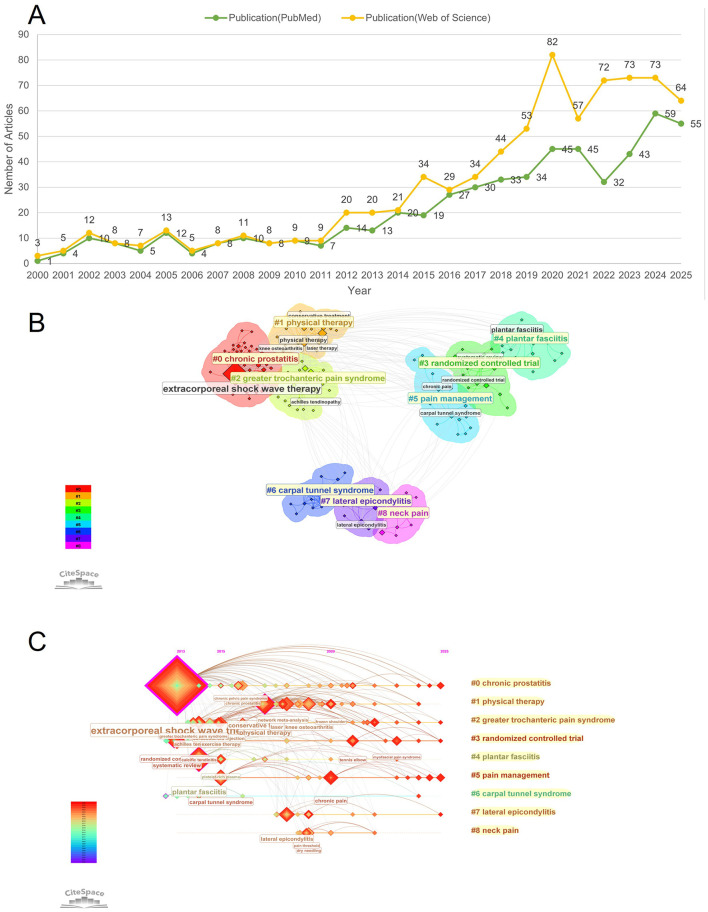
Inter-database verification of evolutionary trends and topical landscapes. **(A)** Comparative distribution of yearly academic outputs across WoSCC and PubMed (2000–2025); **(B)** Dominant research themes derived from PubMed keyword clustering, illustrating the primary focuses within the domain. Distinctive colors signify different thematic clusters, each identified by its pre-dominant keyword label. **(C)** Temporal evolution of keyword clusters. Each color-coded line denotes a specific research trajectory, with its horizontal axis reflecting the chronological duration of the theme. Node dimensions correspond to the prevalence of the respective keywords.

We then compared annual publication trends between WoSCC and PubMed (Figure 9A). Both datasets showed highly parallel growth trajectories, indicating that ESWT-related publications in pain research have increased substantially over the past two decades. Publications indexed in WoSCC peaked in 2020 with 82 articles, whereas those indexed in PubMed peaked in 2024 with 59 articles. The consistency of these trends suggests that the expansion of ESWT research in pain management is a genuine and reproducible phenomenon, rather than an artifact of database-specific coverage.

Comparative keyword analysis further showed that WoSCC and PubMed shared core themes while providing complementary perspectives on ESWT for pain management (Figures 9B, C). In both databases, “extracorporeal shock wave therapy” emerged as a central topic, with shared emphasis on common musculoskeletal pain conditions, including plantar fasciitis, shoulder disorders, suprapatellar tendinopathy, and knee osteoarthritis. Both databases also highlighted clinical research themes, particularly randomized controlled trials and pain management. However, distinct differences were observed. WoSCC contained more mechanistic and interdisciplinary terms, including tendinopathy, chronic pelvic pain syndrome, and myofascial pain syndrome, together with related basic research perspectives. In contrast, PubMed placed greater emphasis on clinically defined conditions, such as chronic prostatitis, neck pain, and carpal tunnel syndrome, as well as practice-oriented aspects of physical therapy. These differences are consistent with the inherent scopes of the two databases: WoSCC offers broader coverage of interdisciplinary and international research, whereas PubMed is more closely aligned with clinical diagnosis, treatment, and practice-based concerns. Overall, the complementary coverage of WoSCC and PubMed strengthens the comprehensiveness and reliability of this study's findings.

## 4 Discussion

Over the past two decades, the annual number of publications regarding ESWT for pain management has increased steadily. This trend aligns with the rising global burden of chronic musculoskeletal disorders and the gradual shift toward non-invasive treatment strategies in clinical practice (20, 21). Our bibliometric data indicate that this field has undergone a phase of slow accumulation (2000–2014) followed by a phase of rapid expansion (2015–2025). This growth in research output has coincided with an increased clinical demand for non-opioid alternatives for pain management amid the global opioid crisis (21). At the same time, organizations such as the International Society for Medical Shock Wave Therapy (ISMST) have successively published standardized ESWT treatment protocols, in tandem with ongoing advances in related research (19, 22, 23). Highly cited literature indicates that ESWT has been increasingly discussed as a physical therapy modality for tendon and soft-tissue disorders, beyond its earlier association with lithotripsy-derived applications (24–26). In this context, the relationship between ESWT research and physiotherapy-oriented practice deserves further attention. From a journal-source perspective, however, physiotherapy-specific journals were not prominent among the core publication and citation sources identified in this bibliometric analysis. The main publication and citation sources were primarily orthopedic, sports medicine, rehabilitation, and general medical journals. This pattern may reflect both the indexing coverage of the selected databases and the historical concentration of ESWT research in orthopedic and sports medicine journals, and should not be interpreted as indicating limited clinical relevance of ESWT to physiotherapy practice.

In terms of geographical distribution and academic influence, a clear disparity was observed between publication volume and citation impact. Although China was the most productive country in this field, with 507 publications, bibliometric indicators of academic influence, including average citations per paper and the H-index, showed that Germany and the United States continued to exert greater academic influence. This pattern may be explained by the high-quality randomized controlled trials (RCTs) conducted by European research teams during the early development of ESWT, which established a solid evidence base for its clinical application. For example, the pioneering studies by Gerdesmeyer et al. (27) and Rompe et al. (28) provided Level I clinical evidence for ESWT in the treatment of plantar fasciitis and Achilles tendinopathy, respectively. The treatment standards established by these studies continue to serve as important references and may partly explain the sustained citation impact of related literature.

Keyword clustering and burst analysis reveal that the focus of research has shifted over time from symptom relief to tissue repair and regeneration. Early studies focused mainly on specific anatomical indications, including plantar fasciitis (28–31), lateral epicondylitis (32–34), and calcific tendinitis (35–37), with the primary aim of validating the analgesic efficacy of ESWT . More recently, the prevalence of keywords including “regeneration,” “mesenchymal stem cells,” and “platelet-rich plasma” has increased markedly across publications, aligning with growing insights into the biological mechanisms of ESWT (38–43).

As described in the review by Wang CJ (24), the mechanism of ESWT is no longer considered solely a process of mechanically fragmenting diseased tissue. Instead, ESWT is increasingly understood as a therapeutic stimulus that promotes local angiogenesis and tissue remodeling through mechanotransduction. This shift suggests that the current frontier of ESWT research lies in its potential synergy with regenerative biologics, with research objectives expanding from short-term pain relief to long-term tissue repair.

The continuous expansion of clinical indications represents another important trend identified in this study. Citation network analysis showed that tendinopathy remains a central focus of ESWT research (5, 44), while interest in more complex conditions, such as knee osteoarthritis (KOA) (6, 12, 13) and chronic pelvic pain syndrome (45–47), has increased rapidly. In recent years, KOA has emerged as a high-frequency keyword, consistent with population aging and the growing clinical demand for conservative treatments for degenerative joint diseases (48). This expansion of indications suggests that future research should further optimize ESWT energy flux density, treatment intervals, and protocol design for deep and complex anatomical structures, such as the knee joint. Such efforts may help establish standardized treatment protocols that differ from those used for superficial tendon disorders.

Nevertheless, this study has several limitations. First, although WoSCC provides standardized citation information and is widely used in bibliometric studies, the use of WoSCC as the primary data source, with PubMed as a validation database, may still have led to the underrepresentation of studies indexed in other sources, such as Scopus, Embase, CINAHL, PEDro, SPORTDiscus, Google Scholar, regional databases, and emerging bibliographic platforms. Therefore, future bibliometric studies incorporating broader bibliographic sources and multi-database search strategies may provide a more comprehensive overview of ESWT-related research in pain management. Second, only English-language publications were included, which may have led to the exclusion of relevant studies published in other languages. Third, because bibliographic databases are updated periodically, the most recent studies may not have been fully captured, potentially delaying the identification of emerging research trends. Despite these limitations, our findings provide useful guidance for scientific planning and the identification of future research priorities in this field. Future studies could combine bibliometric analysis with traditional review methods to examine the key topics identified in this study in greater depth.

Cross-validation using PubMed further strengthened the credibility of our findings. Although the absolute number of publications differed between the two databases, likely because the WoSCC provides broader interdisciplinary coverage whereas PubMed focuses more on clinical literature, their core findings were highly consistent. Regarding annual publication trends, both datasets showed synchronized upward trajectories, confirming that research activity on ESWT for pain management has increased steadily over the past two decades. This pattern suggests that the observed growth is not an artifact of the inclusion characteristics of a single database, but rather reflects a genuine and stable trend in the field. Keyword comparisons also showed substantial consistency and complementarity between the two databases. Both WoSCC and PubMed focused on core topics such as extracorporeal shock wave therapy, plantar fasciitis, shoulder pain, suprapatellar tendinitis, and knee osteoarthritis, reflecting the dominant role of musculoskeletal pain in this research area. Meanwhile, WoSCC placed greater emphasis on mechanistic exploration, specific pathological conditions, and basic research, whereas PubMed focused more on clinically defined disease entities, diagnostic and therapeutic practices, and application-oriented content. This distribution is consistent with the respective scopes of the two databases. Overall, the cross-database consistency supports our conclusion that the bibliometric mapping reflects the true academic evolution of the field rather than database-specific bias.

## 5 Conclusions

This bibliometric analysis provides a comprehensive overview of global research on extracorporeal shock wave therapy (ESWT) for pain management from 2000 to 2025. The findings indicate that this field has entered a stage of consolidation and translational expansion, characterized by sustained growth in publication output, increasingly diversified clinical indications, and a gradual shift from efficacy validation toward mechanistic investigation and regenerative applications. Musculoskeletal pain, particularly plantar fasciitis, shoulder disorders, tendinopathy, and knee osteoarthritis, remains the central research focus, while chronic pelvic pain syndrome and myofascial pain syndrome represent emerging areas of interest.

Although China contributed the largest number of publications, citation-based indicators suggest that Germany, the United States, and other Western countries continue to exert substantial academic influence, largely reflecting their early contributions to high-quality clinical evidence. Cross-database validation with PubMed further confirmed that the major publication trends and research themes identified in the WoSCC dataset were robust and not attributable to database-specific bias. Future research should focus on multicenter randomized controlled trials, standardized and individualized treatment protocols, deeper exploration of mechanotransduction mechanisms, and stronger international collaboration. These efforts will be essential for refining the clinical application framework of ESWT and strengthening its role as a non-pharmacological strategy in multidisciplinary pain management.

## Data Availability

The raw data supporting the conclusions of this article will be made available by the authors, without undue reservation.
