# Supplementary material for: Application of extracorporeal shock wave therapy in pain management: a bibliometric analysis of current advances and trends
Source: Front Med (Lausanne). 2026 Jul 17;13:1867854. doi: 10.3389/fmed.2026.1867854 (PMC13423949; doi:10.3389/fmed.2026.1867854)
Supplement: Supplementary file 1 [file Supplementary_file_1.docx]

**Supplementary Table S1**

**Standard name list of the top 20 authors**

| **Standardized Name** | **Original Variants Found** |
| --- | --- |
| Rompe, Jan D | Rompe, Jan D; Rompe, J; Rompe, JD; Rompe, Jan; Rompe, J. D.; Rompe, Jan-Dirk; Rompe JD |
| Maffulli, Gayle | Maffulli, Gayle; Maffulli, G; |
| Iuliano, Enzo | Iuliano, Enzo; Iuliano, E; |
| Padulo, Johnny | Padulo, Johnny; Padulo, J; |
| Schmitz, Christoph | Schmitz, Christoph; Schmitz C |
| Furia, John P | Furia JP.; Furia JP; Furia, JP; Furia John P; Furia, J; Furia, John; Furia J. P.; |
| Cacchio, Angelo | Cacchio, Angelo; Cacchio, A |
| Gerdesmeyer, Ludger | Gerdesmeyer, Ludger; Gerdesmeyer, L |
| Vulpiani, Maria Chiara | Vulpiani, Maria Chiara; Vulpiani, M. C. |
| Trischitta, Donatella | Trischitta, Donatella; Trischitta, D. |
| Engebretsen, Kaia Beck | Engebretsen, kaia b.; Engebretsen, Kaia Beck |
| Nusca, Sveva Maria | Nusca, Sveva Maria; Nusca, S. M. |
| Santilli, Valter | Santilli, Valter; Santilli, V |
| Schmitt, Jan | Schmitt, Jan; Schmitt, J. |
| Ma, Junming | Ma, jun-ming |
| Maier, Markus | maier, m; Maier, Markus; Maier, M. |
| Mior, Silvano | Mior, Silvano; Mior, Silvano a. |
| Paoloni, Marco | Paoloni, Marco; Paoloni, M |
| Randhawa, Kristi | Randhawa, Kristi; Randhawa, K |
| Respizzi, Stefano | Respizzi, S; Respizzi, Stefano; Respizzi, S. |

**Supplementary Table S2**

**Standard name list of the top 20 organizations**

| **Standardized Name** | **Original Variants Found** |
| --- | --- |
| University of Health Sciences | Univ Hlth Sci; Hlth Sci Univ; Univ Hlth Sci Turkey |
| University Roma La Sapienza | Univ Roma La Sapienza; Sapienza Univ Rome; Sapienza Univ; Univ Rome Sapienza; Univ Rome La Sapienzavia |
| University Bari Aldo Moro | Univ Bari Aldo Moro; Univ Bari; Univ Study Bari |
| University Catanzaro Magna Graecia | Univ Catanzaro Magna Graecia; Magna Graecia Univ Catanzaro; Univ Catanzaro |
| Carol Davila University Medical & Pharmacy | Carol Davila Univ Med & Pharm; Carol Davila Univ Med & Pharm Bucharest; Univ Med & Pharm Carol Davila; Univ Med & Pharm Carol Davila Bucuresti |
| Oslo University Hospital | Oslo Univ Hosp; Oslo Univ Hosp Hf |
| Chengdu University Traditional Chinese Medicine | Chengdu Univ Tradit Chinese Med; Hosp Chengdu Univ Tradit Chinese Med |
| University Schleswig Holstein | Univ Schleswig Holstein; Univ Klinikum Schleswig Holstein; Univ Schlswig Holstein |
| Johannes Gutenberg University Mainz | Johannes Gutenberg Univ Mainz; Univ Mainz |
| Sun Orthopedic & Sports Medical | Sun Orthoped & Sports Med; Sun Orthopaed & Sports Med |
| Canadian Memorial Chiropract College | Canadian Mem Chiropract Coll; Canadian Mem Chiropract College; Canadian Mem Chiropract Coll Cmcc |
| Kanuni Sultan Suleyman Training & Research Hospital | Kanuni Sultan Suleyman Training & Res Hosp; Istanbul Kanuni Sultan Suleyman Training & Res Ho; Kanuni Training & Res Hosp |
| Kaohsiung Municipal Hsiaokang Hospital | Kaohsiung Municipal Hsiaokang Hosp; Kaohsiung Municipal Siaogang Hosp |
| Orthopaedic Klin | Orthopad Klin; Orthopad Klin Kassel; Othopaed Klin Kassel |
| University Roma Tor Vergata | Univ Roma Tor Vergata; Tor Vergata Univ |
| Sun Orthopaedic Group | Sun Orthopaed Grp; Sun Orthopaed Grp Inc |
| Ankara Yildirim Beyazit University | Ankara Yildirim Beyazit Univ; Yildirim Beyazit Univ |
| Daegu Catholic University | Daegu Catholic Univ; Catholic Univ Daegu |
| Hunan University Chinese Medicine | Hunan Univ Chinese Med; Hunan Univ Tradit Chinese Med |
| Karatay University | Karatay Univ; Kto Karatay Univ |

**Standard name list of the top 20 keywords**

**Supplementary Table S3**

| **Standardized Name** | **Original Variants Found** |
| --- | --- |
| Extracorporeal Shock Wave Therapy | Extracorporeal Shock Wave Therapy; Shock Wave Therapy; Extracorporeal Shockwave Therapy; Wave Therapy;Eswt; Shock Wave; Shock Waves; Extracorporeal Shock-Wave Therapy |
| Plantar Fasciitis | Plantar Fasciopathy; Fasciitis Plantaris; Fasciopathy; Chronic Plantar Fasciitis |
| Corticosteroid Injection | Corticosteroid Injection; Corticosteroid Injections; Steroid Injection; Local Corticosteroid Injection |
| Randomized Controlled Trial | Randomized Controlled-Trial; RCT; Randomized Clinical Trial; Randomised Clinical Trial; Randomized Controlled Trial |
| Conservative Treatment | Conservative Treatment; Conservative Therapy |
| Outcome | Outcome; Outcm |
| Meta-Analysis | Meta-Analysis; Metaanalysis; Systematic Review And Meta-Analysis |
| Lateral Epicondylitis | Lateral Epicondylitis; Tennis Elbow; Chronic Tennis Elbow; Epicondylitis Humeri Radialis; Chronic Lateral Epicondylitis; Elbow Tendinopathy; Epicondylitis |
| Calcific Tendinitis | Calcific Tendinitis; Calcifying Tendinitis; Chronic Calcifying Tendinitis; Calcific Tendinopathy; Callcific Tendinopathies; Calcareous Tendinitis |
| Pain Management | Pain Management; Management |
| Heel Pain | Heel Pain; Painful Heel |
| Therapy | Therapy; Intervention |
| Efficacy | Efficacy; Clinical Efficacy; Treatment Effect; Effectiveness; |
| Women | Women; Female; Woman |
| Quality Of Life | Quality Of Life; Quality‑Of‑Life; QOL; Health-Related Quality of Life; |
| Follow Up | Follow Up; Follow-Up |
| Knee Osteoarthritis | Knee Osteoarthritis; Koa |
| Symptoms | Symptoms; Symptom; Clinical Symptoms; |
| Chronic Pelvic Pain Syndrome | Chronic Pelvic Pain Syndrome; Pelvic Pain Syndrome |
| Myofascial Pain Syndrome | Myofascial Pain Syndrome; Myofascial Pain Syndrome (Mps) |

**Standard name list of the top 20 countries**

**Supplementary Table S4**

| **Standardized Name** | **Original Variants Found** |
| --- | --- |
| China | TAIWAN; PEOPLES R CHINA |
| United States | USA; U.S.A.; United States of America |
| South Korea | South Korea; Korea; Republic of Korea |
| Germany | Germany; Republic of Germany |
| Australia | Australia; Commonwealth of Australia; |
| Brazil | Brazil; Brasil |
| United Kingdom | United Kingdom; UK; U.K.; England; Scotland; Wales; Northern Ireland |
| Israel | Israel |
| Italy | Italy |
| Portugal | Portugal |
| Iran | Iran; Islamic Republic of Iran; |
| Spain | Spain |
| Canada | Canada |
| New Zealand | New Zealand |
| Turkey | Turkey; Turkiye; |
| Japan | Japan |
| Switzerland | Switzerland; Swiss Confederation; |
| Sweden | Sweden; Kingdom of Sweden; |
| Denmark | Denmark; Kingdom of Denmark; |
| Norway | Norway; Kingdom of Norway; |
